# Nocturnal Fanning Suppresses Downy Mildew Epidemics in Sweet Basil

**DOI:** 10.1371/journal.pone.0155330

**Published:** 2016-05-12

**Authors:** Yigal Cohen, Yariv Ben-Naim

**Affiliations:** The Mina &Everard Goodman Faculty of Life Sciences, Bar Ilan University, Ramat-Gan, Israel; Leibniz-Institute of Vegetable and Ornamental Crops, GERMANY

## Abstract

Downy mildew is currently the most serious disease of sweet basil around the world. The oomycete causal agent *Peronospora belbahrii* requires ≥ 4h free leaf moisture for infection and ≥7.5h of water-saturated atmosphere (relative humidity RH≥95%) at night for sporulation. We show here that continued nocturnal fanning (wind speed of 0.4–1.5 m/s) from 8pm to 8am dramatically suppressed downy mildew development. In three experiments conducted during 2015, percent infected leaves in regular (non-fanned) net-houses reached a mean of 89.9, 94.3 and 96.0% compared to1.2, 1.7 and 0.5% in adjacent fanned net-houses, respectively. Nocturnal fanning reduced the number of hours per night with RH≥95% thus shortened the dew periods below the threshold required for infection or sporulation. In experiments A, B and C, the number of nights with ≥4h of RH≥95% was 28, 10 and 17 in the non-fanned net-houses compared to 5, 0 and 5 in the fanned net-houses, respectively. In the third experiment leaf wetness sensors were installed. Dew formation was strongly suppressed in the fanned net-house as compared to the non-fanned net-house. Healthy potted plants became infected and sporulated a week later if placed one night in the non-fanned house whereas healthy plants placed during that night in the fanned house remained healthy. Infected potted basil plants sporulated heavily after one night of incubation in the non-fanned house whereas almost no sporulation occurred in similar plants incubated that night in the fanned house. The data suggest that nocturnal fanning is highly effective in suppressing downy mildew epidemics in sweet basil. Fanning prevented the within-canopy RH from reaching saturation, reduced dew deposition on the leaves, and hence prevented both infection and sporulation of *P*. *belbahrii*.

## Introduction

Downy mildew caused by the oomycete *Peronospora belbahrii* is a devastating disease of sweet basil around the world [1]. The pathogen attacks basil leaves causing chlorosis followed by necrosis and defoliation making it unmarketable. First appearance of the disease in Israel occurred in 2011 and quickly became epidemic in all growing areas in the country [2]. A year after its first appearance in Israel, the pathogen developed resistance to mefenoxam, thus making one of the most effective registered fungicide, ineffective[2].Currently, dimethomorph and azoxystrobin are used for the control of the disease. The pathogen propagates by asexual spores produced at night on the surface of infected leaves under humid conditions. The spores discharge, germinate in the presence of water, and infect healthy leaves. New spores are produced within a week. In a previous paper [3] we demonstrated the effective control of the disease by nocturnal illumination which suppresses sporulation of the pathogen on the leaf surfaces. In a later study [4] we showed that spores *P*. *belbahrii* and its mycelium colonizing basil leaves are sensitive to extreme heat. Exposure of spores, infected leaves, or infected plants to 35–45°C for 6–9 hours suppressed pathogen’s survival. Day-time solar heating achieved by covering basil crops growing in net-houses with polyethylene sheets was highly effective in suppressing disease development and increasing yield [4]. Here we report on yet another measure that can help prevent the pathogen from attacking basil: fanning the crops at night. Our working hypothesis was that such fanning will reduce relative humidity and dew deposition and hence will prevente infection and sporulation.

## Materials and Methods

### Plants

The sweet basil (*Ocimum basilicum*) cultivar Peri (Volcani Center for Agricultural Research, Newe Ya’ar, Israel) was used in all experiments. Plants were grown in 0.5l pots filled with peat: vermiculite (1:1, v/v) in the greenhouse (night/day temperature 18°C/32°C). Plants were used for experiments at the 10–12 leaf stage, unless stated otherwise.

### Net-houses and nocturnal fanning

The effect of nocturnal fanning on epidemics of basil downy mildew incited by *P*. *belbahrii* were tested in three successive experiments A, B and C, conducted in different time periods during 2015 at Bar-Ilan University Farm (32° 4' 9" N / 34° 50' 35" E). Basil plants cultivar Peri at the 4-leaf stage were planted in 120×50×22 cm polystyrene containers filled with peat: perlite: vermiculite (1;1;1, v/v) mixture, 18 plants per container. Containers were arranged in 3 rows, 1m apart, 25 containers in a row, inside two 50x6m walk-in net-houses, non-fanned (NH 5) and fanned (NH 6). Net-houses were covered with a white, 50-mesh insect-proof plastic net. They were oriented at west-east direction. Six fans (50 cm diameter) were installed in NH 6, 7.2m apart, above the middle row at 1.5 m above ground, inclined 30 degrees toward the ground. Fans were operated nightly from 8pm to 8am (nocturnal fanning) all along the course of each experiment. Wind speed was measured at plant canopy level, 50 cm above ground, using WindSonic 2-Axis Ultrasonic Anemometer (Gill Instruments, Lymington, Hants, UK) equipped with CR 3000 micrologger (Campbell Scientific, Shepshed, UK). Temperature and relative humidity were monitored within the canopy by using HOBO data logger UX100-003 (Onset ComputerCorp. USA). Leaf wetness in Experiment C was measured using Hobo S-LWA-M003 sensors, equipped with Hobo H21-002 data logger.

Planting basil for Experiments A, B and C took place on June 1 to June 24, August 10 to September 7, and September17 to October 14, 2015, respectively. Plants were drip-irrigated 4 times (15 min) a day. Twice a week plants were fertigated with 0.5% N;P:K (20:20:20) solution. No pesticides were applied.

Two days after planting, sporulating potted basil plants were transplanted as inoculum spreaders, one plant per container, to allow the establishment of the disease in the newly planted crops. Spreader plants were removed a week later. This procedure of planting spreader plants was done in all 3 experiments. Disease records were taken periodically (see below) by counting the total number of leaves and the number of sporulating leaves in each container.

In each experiment, 80 healthy potted plants were incubated in a growth chamber at 25°C for 2 weeks to assure their freedom from the disease. At the end of this period no plant has shown downy mildew symptoms

At the end of each experiment plants were uprooted and destroyed. Fresh soil mixture was added to containers before a new planting took place.

### Pathogen, inoculation, colonization and sporulation

The mefenoxam-resistant isolate Knafo 3 of *P*. *belbahrii* was used in all experiments. This isolate was collected in southern Israel in 2013. Plants were inoculated by spraying a spore suspension (5000 spores/ml) on their upper leaf surfaces until run off. Inoculated plants were kept in a dew chamber (Percival Scientific, Perry, IA, USA) at about 100%RH (18°C) in the dark for 20h (unless stated otherwise) to promote infection and then transferred to a growth chamber at 25°C (50–60%RH) with continuous illumination (cool white and warm white fluorescent lamps, 60 μmole m^2^ s^-1^) to allow for pathogen colonization but not sporulation. At 6 dpi (days post inoculation) plants, exhibiting chlorotic lesions on their leaves, were returned to the dew chamber for 20h (unless stated otherwise) to induce sporulation of the pathogen. Heavy sporulation occurred on the lower leaf surfaces, more on upper (younger) than on the lower (older) leaves. Percent leaf area occupied by spores was visually estimated and served to determine the effect of the various treatments employed (see below) on disease.

### Effect of fanning on infection and sporulation

To determine whether fanning can prevent infection, 80 healthy potted basil plants at 12–leaf stage were placed between the rows of sporulating plants in the non-fanned net-house for 4 hours starting at 8am in order to expose them to spore shower. At 12 noon, half of the plants were transferred into the non-fanned house. Both groups of plants were returned to the laboratory after 24h, placed in a dew chamber for 12h to allow infection, incubated at 25°C for 6 days for symptom production and then placed in a dew chamber to allow sporulation.

To test the effect of fanning on sporulation, 80 healthy potted basil plants at 12-leaf stage were inoculated in the laboratory with *P*. *belbahrii*. At 6 dpi 40 plants were placed for one night in the fanned net-house and 40 plants in the non-fanned house. Percent leaf area sporulating in the following morning was visually estimated.

### The effect of temperature and dew period on infection

Potted plants at the 10–12 leaf stage were spray-inoculated with spores of *P*. *belbahrii* and placed in a dew chamber at various temperatures for 2–24h. Plants were then incubated at 25°C as stated above to allow for pathogen colonization. At 6 dpi they were returned to a dew chamber at 20°C in the dark to allow the pathogen to sporulate. Percent leaf area occupied by spores was visually estimated and served to determine the effect of temperature and dew period duration on infection.

### The effect of RH, temperature and dew period on sporulation

Detached infected leaves of basil (6 dpi) hanged over saturated salt solutions inside sealed darken boxes at 20°C for 24h were used to measure the effect of RH on sporulation [4]. Infected potted plants at 6 dpi (see above) were wrapped with moistened plastic bags and incubated in the dark at various temperatures for 4–24h. Percent leaf area occupied by spores was visually estimated and served to determine the effect of temperature and dew period duration on sporulation.

### The effect of interrupted dew period on infection and sporulation

Potted basil plants at the 12 leaf stage were inoculated with *P*. *belbahrii* and placed in a dew chamber (20°C, darkness) for 9 hours to allow infection. At one hour intervals, a group of 6 plants was removed from the chamber, fanned (3 m/s) for 10 min at 30°C to dry off the inoculum droplets and returned to the dew chamber. After 9 hours all plants were transferred to 25°C for 6 days. Plants were then allowed to sporulate for 20h at 20°C in the dew chamber and the percent of leaf area sporulating was used to determine the effect of the interruption of the dew period on infection.

A similar approach was undertaken to study the effect of interrupting the dew period on sporulation, except that infected plants at 6 dpi were used.

### Microscopy

To follow the infection process, basil plants at the 12-leaf stage were inoculated with *P*. *belbahrii* and incubated at 15°C in the dark. At 1h time intervals (see Results), leaf discs (12 mm diameter) were removed, clarified in boiling ethanol for 5 min and stained with calcofluor and basic aniline blue and examined with the aid of Olympus A70 epi-fluorescent microscope.

To follow the sporulation process, 12-leaf infected basil plants at 6 dpi were incubated at 18°C in the dark and leaf discs were removed at 1h time intervals (see Results) and processed for microscopic examination as described above.

### Data analysis: field experiments

Three successive plantings (experiments), in June, August and September 2015 were conducted in two adjacent net-houses, a regular, non-fanned net-house and a net-house equipped with 6 fans. Experiments were not replicated and the treatment (fanning) was not randomized. The percentage of sporulating leaves in each net house (75 containers with 18 plants in a container) was recorded periodically and used to calculate the mean± standard deviation of the mean of disease progress in the regular non-fanned house and fanned net-house.

### Data analysis: laboratory experiments

All laboratory experiments were repeated two or more times with 4–6 plants (10 to 12 leaf stage) per treatment. Results from the repeated experiments showed little variation and therefore were combined before analyses. Analysis of variance of laboratory experiments was done using JMP software (SAS Institute). Significant differences between means for α = 0.05 were performed using Tukey’s HSD (honest significant difference) test.

## Results

### Nocturnal fanning

Wind speed in the fanned net-house decreased linearly along the middle row with increasing the distance from the fan, from about 1.5 m/s at a distance of 1m to about 0.7 m/s at a distance of 6 m. Wind speed along the left and right rows decreased from about 1 to 0.4 m/s.

**Fig 1:**
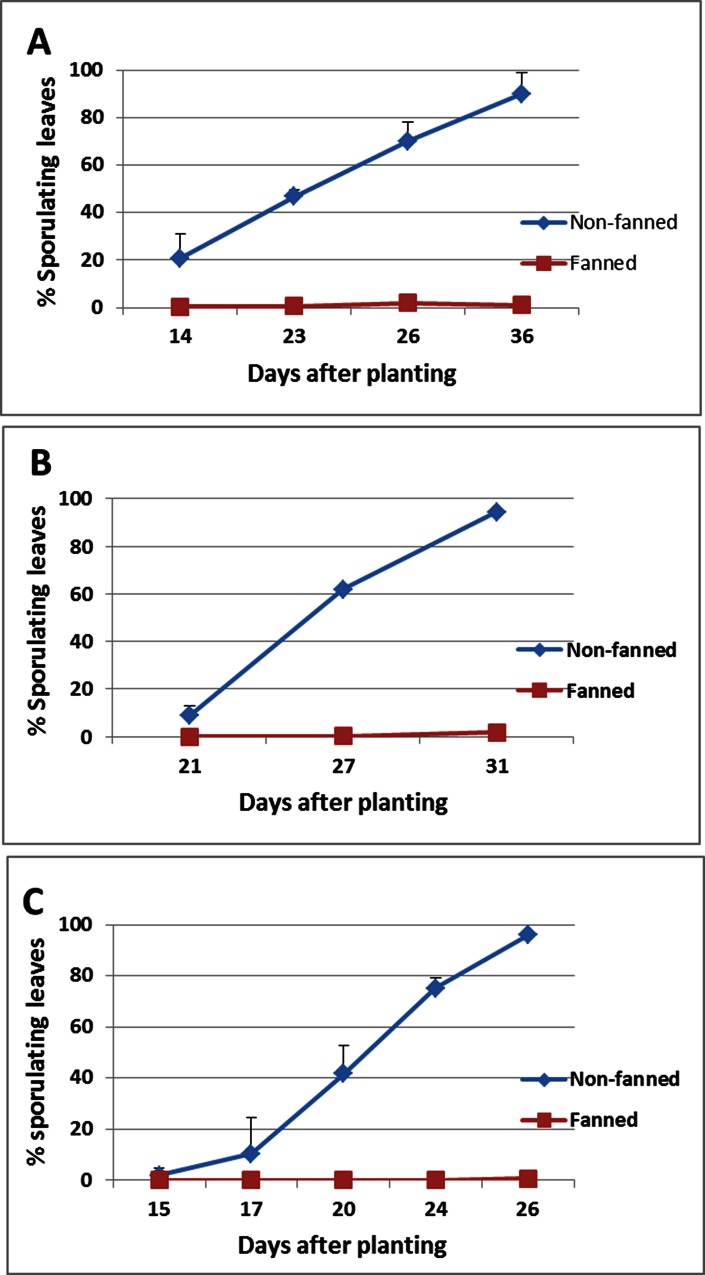
Development of downy mildew in basil crops grown in two net-houses, NH 5 non-fanned and NH 6- fanned (nightly, from 8pm to 8am). **A—**Experiment A- planting on 1.6.2015. **B—**Experiment B planting on 10.8.2015. **C—**Experiment C planting on 17.9.2015.

The epidemic progress of basil downy mildew in Experiments A, B and C is shown in Fig 1. In all three experiments, nocturnal fanning has dramatically suppressed downy mildew development as compared to the non-fanned crops throughout the season. Thus, at the end of Experiment A, B and C, percentage of sporulating leaves in fanned *vs* non-fanned crops were 1.21 *vs*. 89.9%; 1.7 *vs* 94.3%; and, 0.5 *vs*. 96.0%, respectively.

**Fig 2:**
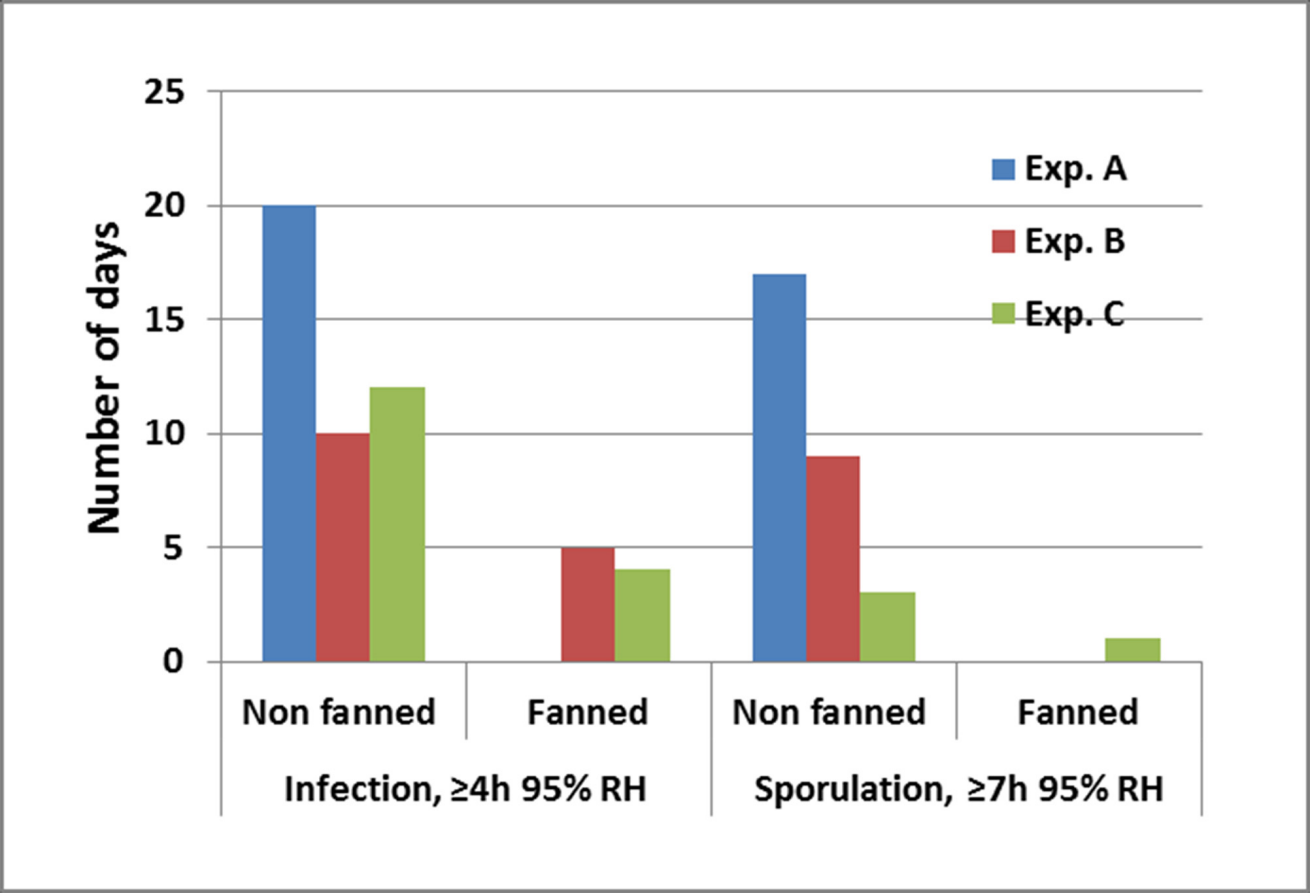
Number of days with conducive conditions for infection (≥4h with RH ≥95%) or sporulation (≥7h with RH ≥95%) for *Peronospora belbahrii* in non-fanned and fanned basil crops in each of three experiments conducted in three growing periods during 2105. Total number was 37, 29, and 28 days in Experiments A, B, and C, respectively.

Based on our controlled experiments (see below) and on a simple empirical model indicating that duration of leaf wetness is equal to the number of hours with RH ≥95% [5] we considered periods of ≥4h with RH ≥95% conducive for infection of basil with downy mildew and periods of ≥7h with RH ≥95% conducive for sporulation of *P*. *belbahrii*. Fig 2 shows that in all three experiments conducing conditions for infection and sporulation were much more frequent in the fanned house as compared to the non-fanned house. For example, in Experiment A, 20 and 17 days out of 37 days were conducive for infection and sporulation, respectively in the fanned house as compared to 0 and 0 days in the non-fanned house.

Plants in both houses were visually scored for dew deposition every morning (except Saturdays) at about 7am. In almost all mornings that dew was observed, leaves in the non-fanned house were wet while leaves in the fanned house were dry.

### Effect of fanning on infection

All 40 potted plants that were inoculated by spore shower and thereafter kept in for one night in the non-fanned house became infected, showing a week later, a mean of 60±10% sporulating leaf area. In contrast, all 40 plants that spent that night in the fanned house showed a week later no infection.

### Effect of fanning on sporulation

All 40 potted infected (6 dpi) plants that were placed for one night in the non-fanned house showed heavy sporulation (mean of 80±10% sporulating leaf area). In contrast, of the other 40 infected plants that spent that night in the fanned house 37 plants showed no sporulation and 3 plants showed 13.3±5.8% sporulating leaf area.

### Effect of dew period and temperature on infection

**Fig 3:**
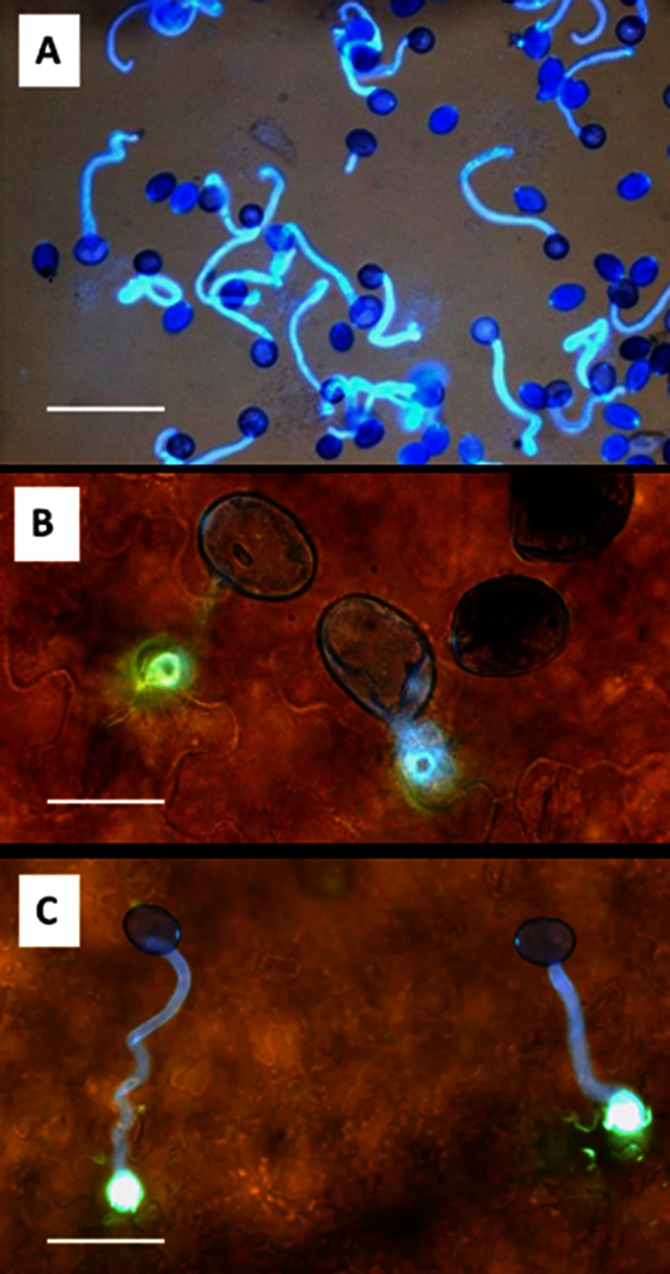
Spore germination and penetration of *Peronospora belbahrii*. **A**- Germination in water, 6h at 15°C in the dark. Bar = 100μm. Calcofluor staing. **B**- Penetration into a basil leaf at 6h at 15°C in the dark. Bar = 25μm. **C**—Penetration into a basil leaf at 24h at 15°C in the dark. Bar = 50 μm. Note in **B** the holes produced by the pathogen in the periclinal walls of the epidermal cells. Note in **C** the callose (yellow) deposits around the penetrated sites. Photos **B** and **C** were taken with the aid of Olympus A70 fluorescent microscope at different field depths after staining with calcofluor and basic aniline blue.

Spores of *P*. *belbahrii* required incubation of ≥2h in water at 15–20°C to germinate (Fig 3A). Free leaf moisture of ≥4h at 15–20°C allowed the germ-tube to penetrate into the leaf epidermis of intact plants (Fig 3B and 3C).

**Fig 4:**
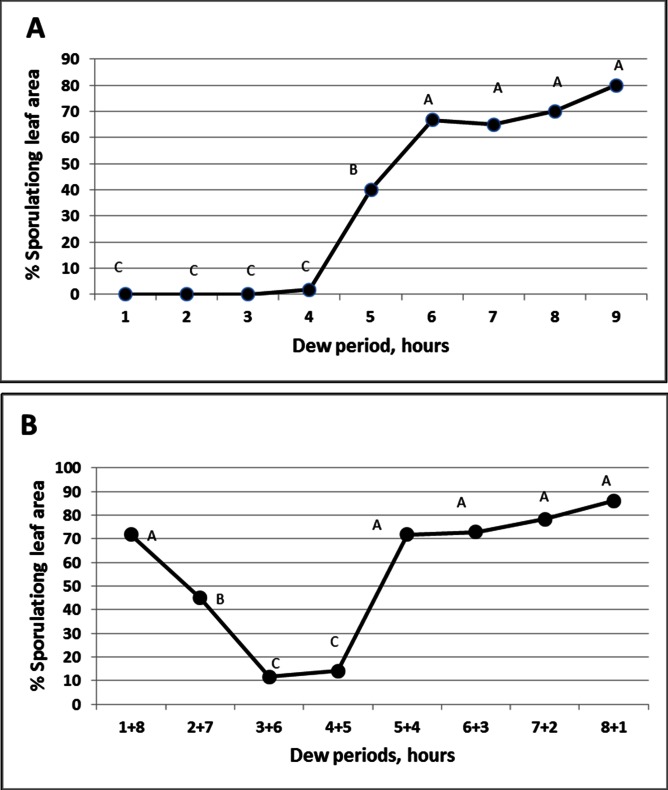
The effect of dew period duration on infection of basil plants with *Peronospora belbahrii*. **A**- Continuous dew period of 1 to 9 hours at 15°C in the dark. **B**- The effect of 10 min dry period during 9h dew period on infection. The 9h dew period was interrupted at 1h time intervals after inoculation by 10 min of dryness, after which the plants were returned to the dew chamber. Different letters in the graphs indicate significant differences between means (Tukey-Kramer HDS analysis, α = 0.05).

Basil plants exposed to spore shower (in the field or in growth chambers) produced no infection unless kept wet (in a dew chamber or sealed in wet plastic bag) after inoculation. The effect of dew period duration on infection at 15°C is shown in Fig 4A. Little infection was seen at 4h of dew period. Infection rate increased significantly at 5h and reached near maximum at 6h. Slight, increase in infection occurred with increasing the dew period to 7–9h.

Interrupting the infection process with a 10 min fanning period had an adverse effect on infection. Fig 4B shows that a 10 min interruption period, employed during the 9 hours dew period (namely, remove the wet inoculated plants from the dew chamber at 1h time intervals after inoculation, dry the leaves by fanning for 10 min at 30°C and return the plants to the dew chamber) had a strong negative impact on infection when applied at 3 or 4h post inoculation, when spore germ-tubes were at their critical stage of penetration (Fig 4B).

**Fig 5:**
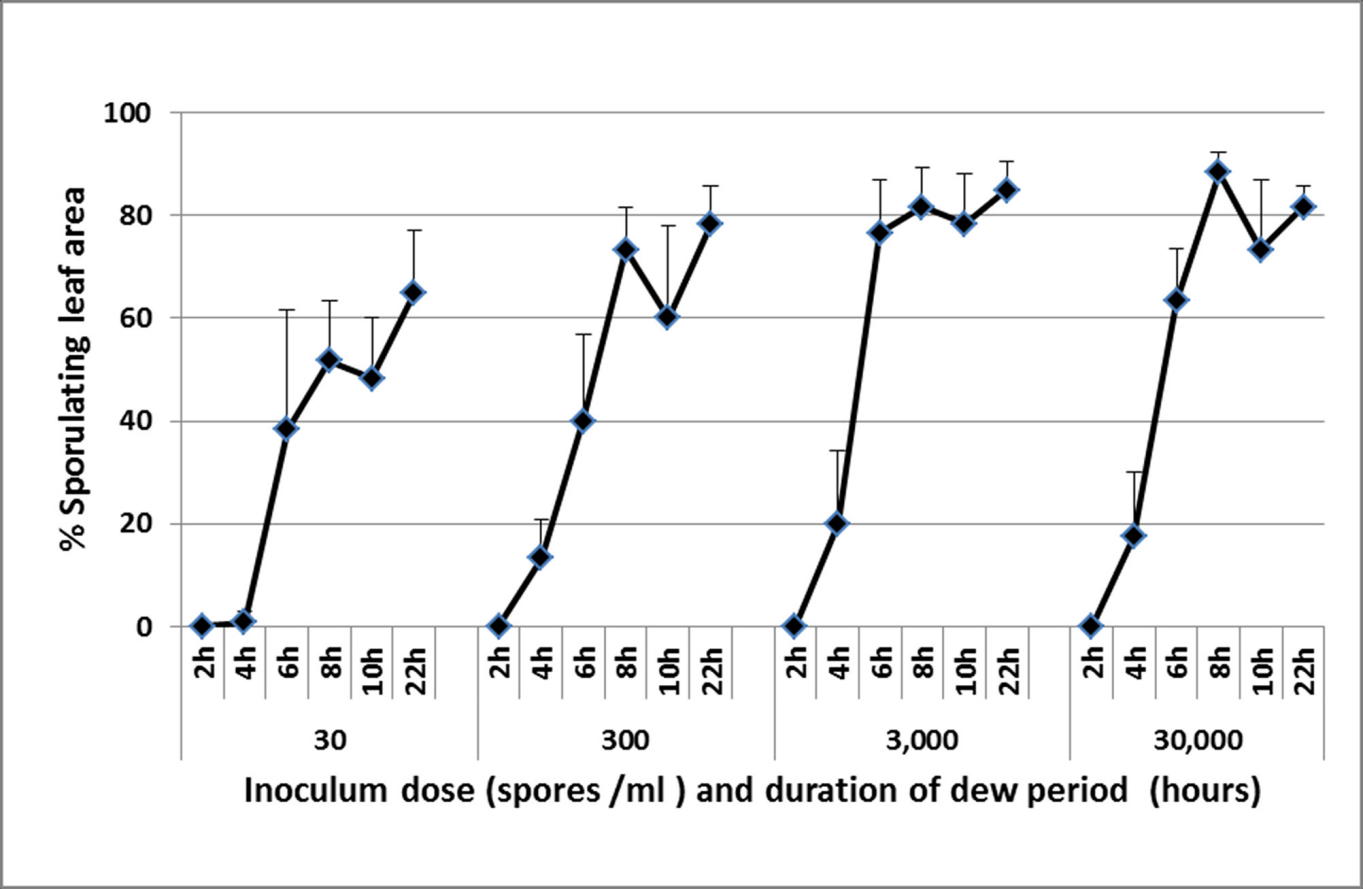
The effect of dew period duration and spore concentration (spores/ml) on infection of basil plants with *Peronospora belbahrii* at 15°C in the dark. Bars indicate the standard deviation of the means.

The effect on infection of dew period duration and inoculum dose (in the dark at 15°C) is shown in Fig 5. At 2h, no infection occurred, regardless of the inoculum dose used. At 4h, infection occurred with ≥300 spores/ml whereas at ≥6h infection occurred at all doses, from 30 to 30,000 spores per ml.

### Effect of RH, dew period and temperature on sporulation

**Fig 6:**
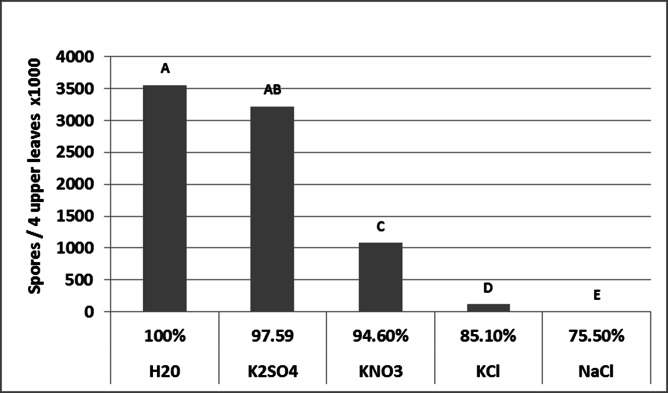
The effect of relative humidity on sporulation of *Peronospora belbahrii* in detached basil leaves. Leaves were kept in sealed boxed over saturated salt solutions for 24h at 20°C in the dark. Different letters on bars indicate significant differences between means (Tukey-Kramer HDS analysis, α = 0.05).

Unlike infection, sporulation did not require free leaf moisture. Data are shown in Fig 6. The number of spores produced decreased as RH decreased. Spore production was equally high at RH 100% and RH 97.59%, but significantly lower at RH 94.60 and almost nullified at RH 85.10%. No sporulation occurred at RH 75.50 (Fig 6).

**Fig 7:**
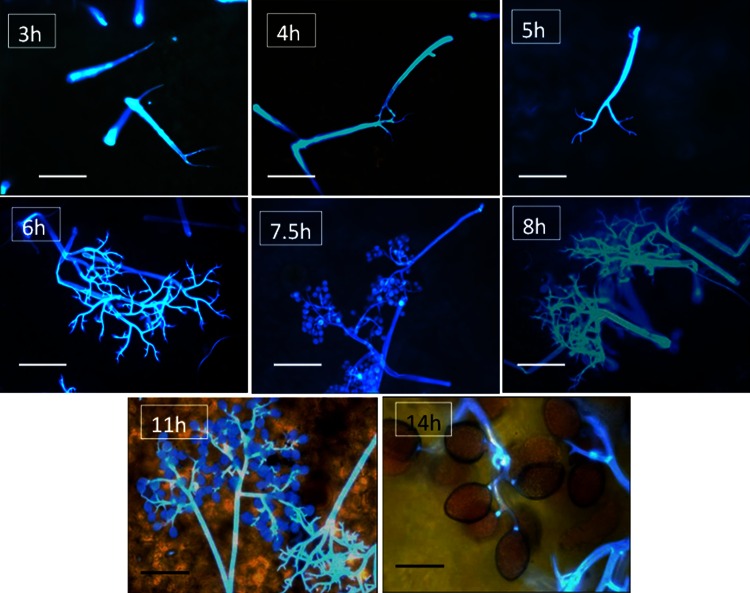
The dynamics of sporulation of *Peronospora belbahrii* on leaf surface of intact basil plants kept in a dew chamber at 18°C in the dark. Photos were taken with the aid of a UV epi-fluorescent microscope after staining with calcofluor (3h–8h) or calcofluor and basic aniline blue (11h and 14h). Bar in 3h-8h = 100μm; Bar in 11h = 50 μm; Bar in 14h = 25 μm.

**Fig 8:**
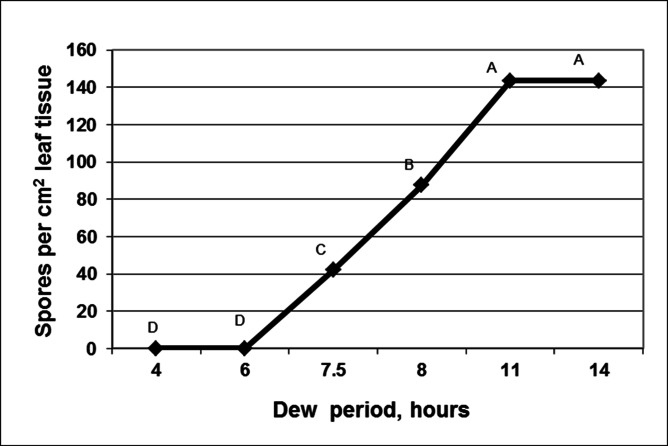
Spore yield of *Peronospora belbahrii* on leaf surface of intact basil plants kept in a dew chamber at 18°C in the dark for 4–14 hours. Different letters in the graphs indicate significant differences between means (Tukey-Kramer HDS analysis, α = 0.05).

The process of sporulation in darkness was followed microscopically in infected basil plants (6 dpi) introduced into a dew chamber in the dark at 18°C (Fig 7). At about 3h, white sporophores start emerging from stomatal openings on the lower leaf surface. At 4–5h, sporophores were branched once or twice. At 6h sporophores were branched trice. At 7.5–8h, spores had developed on the brachlets tips. At 11–14h sporulation reached its final stage with maximal number of spores produced. The effect of dew period duration at 18°C on the number of spores produced per unit leaf area is shown in Fig 8.

**Fig 9:**
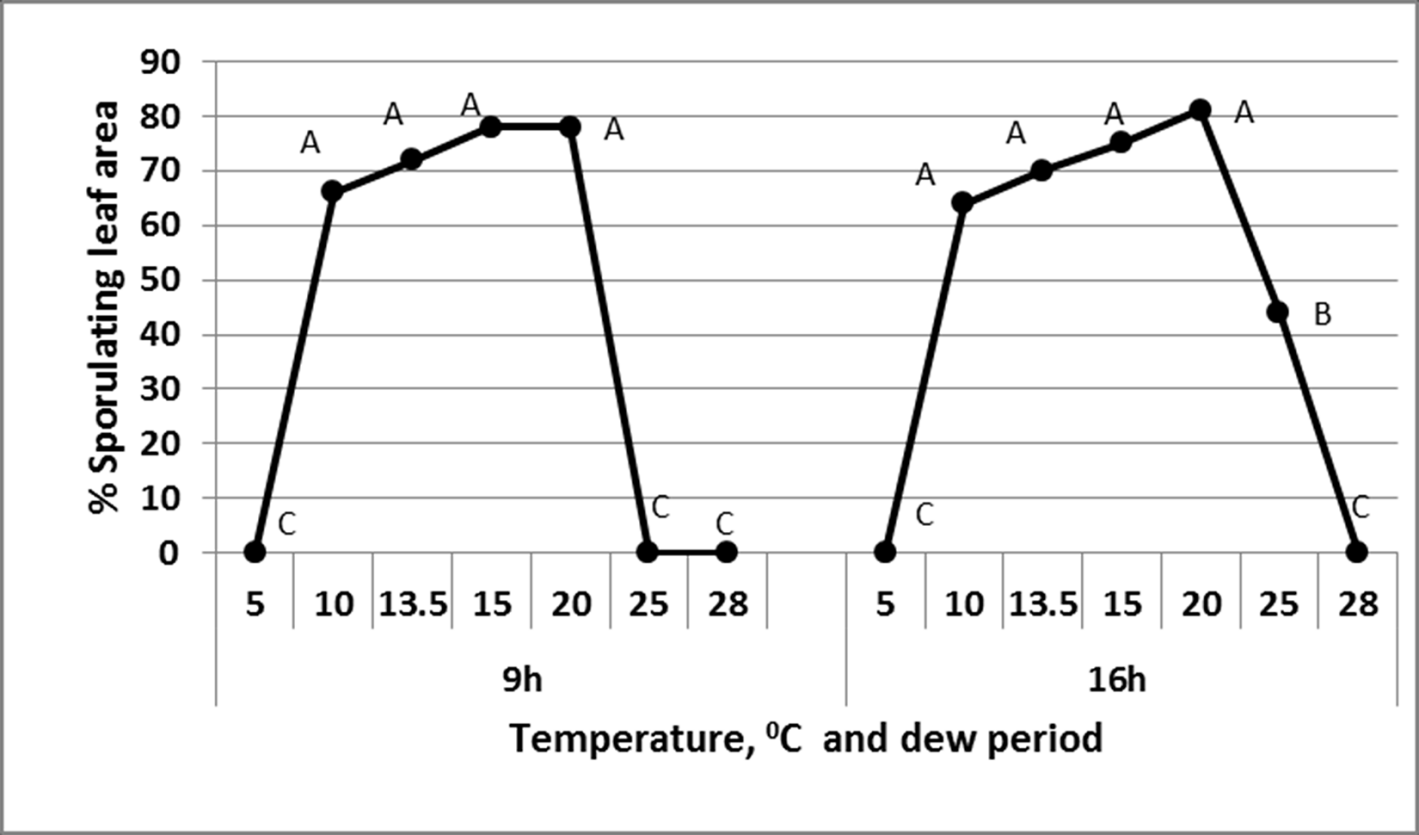
The effect of temperature and moist period duration on sporulation of *Peronospora belbahrii*. Infected basil plants at 6dpi were kept in a dew chamber at 20°C in the dark. Percentage sporulaing leaf area was assessed after 9 or 16 hours of moist period. Different letters in the graphs indicate significant differences between means (Tukey-Kramer HDS analysis, α = 0.05).

The effect of temperature on sporulation in intact basil plants in darkness is shown in Fig 9. When plants were kept in moisture-saturated atmosphere for 9h, sporulation occurred at 10–20°C. When the moist period was extended to 16h, sporulation occurred at 10–25°C. No sporulation occurred at 5°C or 28°C (Fig 9).

**Fig 10:**
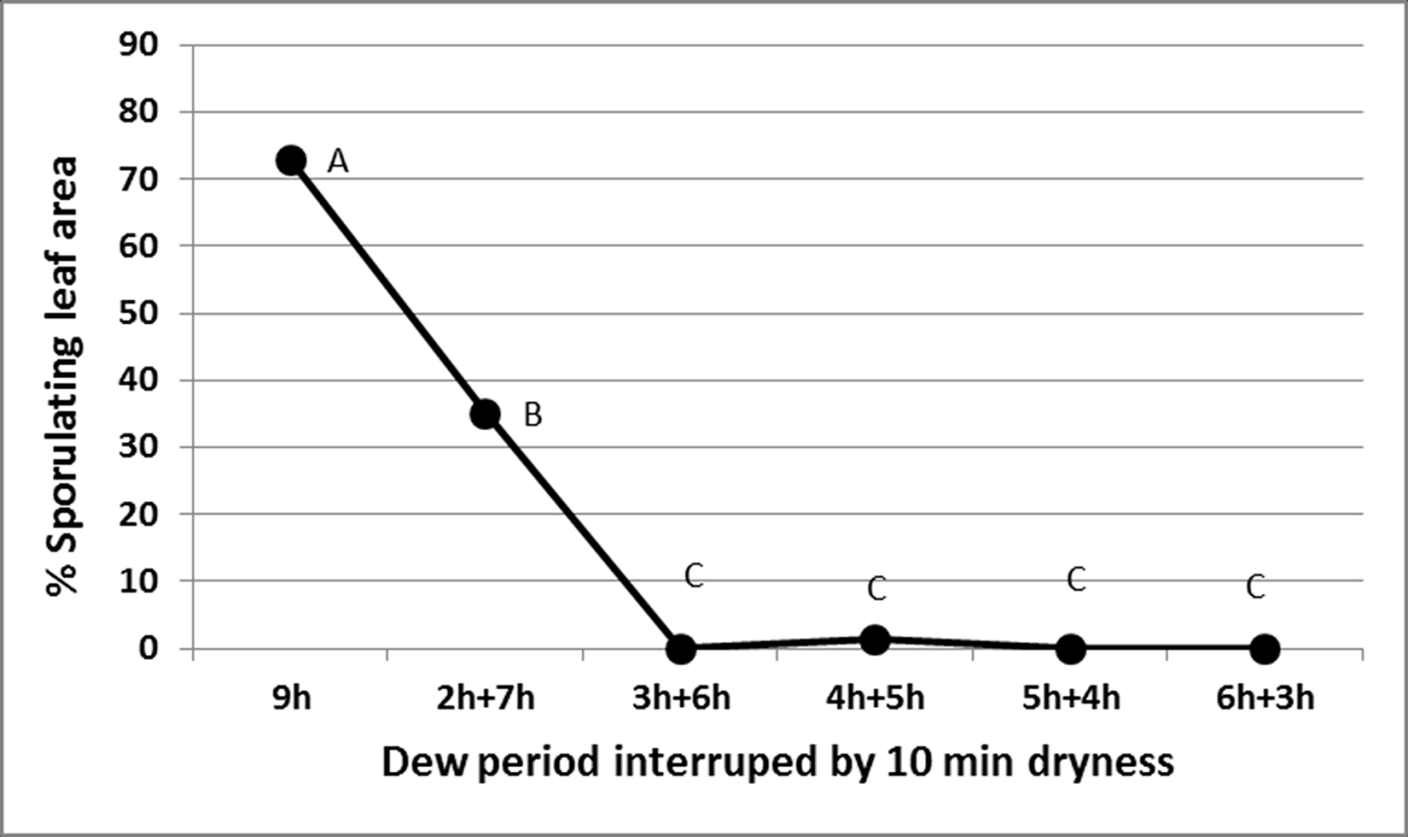
The effect of interrupted dew period on sporulation of *Peronospora belbahrii*. Infected basil plants at 6dpi were kept in a dew chamber at 20°C in the dark. After 2, 3, 4, 5, or 6 hours plants were taken out, fanned for 10 min at 30°C to dry off leaf surface moisture and returned back into the dew chamber. Percentage sporulating leaf area was estimated after 9 hours. Different letters in the graphs indicate significant differences between means (Tukey-Kramer HDS analysis, α = 0.05).

Interrupting the dew period with a 10 min period of drying using fans greatly reduced spore production. Infected basil plants at 6dpi were placed in a dew chamber at 20°C. They were taken out of the chamber at 1h time intervals, fanned (5m/s) for 10 min at 30°C until leaf surfaces dried up and returned to the dew chamber to complete the 9h dew period. Results presented in Fig 10 show that plants kept continuously in the dew chamber for 9h showed sporulation on 73% of their lower leaf surfaces, whereas plants whose dew period was interrupted after 2, 3, 4, 5 or 6h showed sporulation on 35, 0, 1, 0 and 0% of their lower leaf surfaces, respectively (Fig 10). At ≥3h the sporophores have already emerged from stomata; if exposed to dryness they become desiccated and failed to produce spores.

## Discussion

Infection and sporulation of the oomycete foliar plant pathogen *P*. *belbahrii* are strongly dependent on the availability of free moisture and high humidity, respectively. Infection, composed of spore germination and subsequent penetration into the epidermal cells, is shown here to occur within 4h at optimal temperature of 20°C, confirming previous findings [6]. The absolute necessity of free leaf moisture for infection was reported for other foliar downy mildew agents [7–11]. The results we obtained with downy mildew of cucumber showed that dew period duration and inoculum dose compensated for each other during infection [8]. Interrupting the infection process with a 10 min dry period during the first 4 hours reduced infection, probably due to its adverse effect on the developing germ-tubes and/or appressoria.

We show here that sporulation of *P*. *belbahrii* requires high humidity but not free leaf moisture, similar to other foliar downy mildew agents [12–15]. Mature spores were produced on infected plants after incubation of ≥7.5h in humid conditions. Interrupting the sporulation process during the first 6 hours with a 10 min dry period, reduced or abolished subsequent spore formation, probably due to adverse effects on the developing sporophores.

Our working hypothesis was that perturbation of the infection and/or sporulation processes would adversely affect the epidemic progress of downy mildew in basil crops in nature. In Israel, basil is often grown in net-houses. We therefore used nocturnal fanning in net-houses to achieve that goal. We chose to fan during the night when humidity increases and dew is likely to deposit. Indeed, nocturnal fanning applied to basil crops growing in net-houses dramatically suppressed the epidemic build-up of basil downy mildew throughout the growing season. The level of disease control achieved by nocturnal fanning was remarkable, greater than any fungicide we have tested before under similar net-house conditions [16]. Two other physical measures were recently shown to suppress epidemics of downy mildew in basil: nocturnal illumination [3] and day-time solar heating [4]. Both were highly efficient in controlling the disease but inferior to the nocturnal fanning method reported here.

The suppression of the disease resulted from the strong negative effect of fanning on infection and sporulation. We showed here that potted healthy plants became infected when placed for one night in the non-fanned house but not in the fanned house. This suggests that free leaf moisture, available in the non-fanned house, but not in the fanned house, made infection possible. Also, potted infected plants at 6 dpi that spent one night in the non-fanned house showed profuse sporulation in contrast with similar plants which showed almost no sporulation after spending that night in the fanned house, pointing to the negative effect of fanning on sporulation.

Wind has been shown to reduce sporulation of other foliar downy mildew pathogens [11,15]. The effects of temperature (5 to 25°C), relative humidity (81 to 100%), wind speed (0 to 1.0 m/s), and their interactions on sporulation of the downy mildew pathogen *Bremia lactucae* on lettuce cotyledons were investigated in controlled conditions [15]. Sporulation was optimum at 15°C, increasing markedly at RH ≥ 90%. Both RH and wind speed affected sporulation. No sporulation was observed at wind speeds of >0.5 m/s, regardless of RH. In still air, the number of sporangiophores produced per cotyledon increased linearly with RH from 81 to 100%. Similar results were obtained with *Phytophthora infestans* in intact potato plants [11].

Analyses of the meteorological data we collected during our experiments showed minor differences in air temperature between fanned and non-fanned houses while major differences were recorded in relative humidity of ≥95%. In general, relative humidity approached saturation much more frequently and remained so for much longer periods in the non-fanned houses as compared to the fanned houses. This made the environment in the non-fanned houses much more conducive to both infection and sporulation of *P*. *belbahrii* as compared to the fanned houses. As mentioned above, infection requires at least 4h of leaf wetness and sporulation requires at least 7.5 hours of high relative humidity. Such conditions were frequent in non-fanned houses but rare in fanned houses. As a consequent, epidemics progressed very rapidly in non-fanned houses but very slowly in fanned houses.

Wind is known to prevent dew formation in nature [15]. This report confirms that it may happen inside net-houses. Dew is known to deposit on still objects; wind, even at a low speed, disturbs the condensation of water on leaf surface because it removes the saturated air away from the leaf boundary layer [12, 15].

## References

[1] Wyenandt CA, Simon JE, Pyne RM, Homa K, McGrath MT, Zhang S, et al 2015 Basil downy mildew (*Peronospora belbahrii*): Discoveries and challenges relative to its control. Phytopathology 105:885–894. 10.1094/PHYTO-02-15-0032-FI 25894318

[2] Cohen Y, Vaknin M, Ben-Naim Y, Rubin AE, Galperin M, Silverman D, et al 2013 First report of the occurrence and resistance to mefenoxam of *Peronospora belbahrii*, causal agent of downy mildew of basil (*Ocimum basilicum*) in Israel. Plant Disease 97:692. 10.1094/PDIS-12-12-1126-PDN 30722212

[3] Cohen Y, Vaknin M, Ben-Naim Y, Rubin AE (2013) Light suppresses sporulation and epidemics of *Peronospora belbahrii*. Plos One 8: e81282 10.1371/journal.pone.0081282 24348919 PMC3861544

[4] Cohen Y, Rubin AE (2015) Daytime solar heating controls downy mildew *Peronospora belbahrii* in sweet basil. PLoS ONE 10(5):e0126103 10.1371/journal.pone.0126103 25992649 PMC4439122

[5] Rowlandson T, Gleason M, Sentelhas P, Gillespie T, Thomas C, Hornebuckle B, et al (2015) Reconsideration leaf wetness duration determination for plant disease management. Plant Disease 99: 310–319. 30699706 10.1094/PDIS-05-14-0529-FE

[6] Djalali Farahani-Kofoet R, Römer P, Grosch R (2014) Selecting basil genotypes with resistance against downy mildew. Scientia Horticulturae (Amsterdam) 179: 248–255.

[7] Rotem J, Cohen Y, Putter J (1971) Relativity of limiting and optimum inoculum loads, wetting durations, and temperatures for infection by *Phytophthora infestans*. Phytopathology 61: 275–&.

[8] Cohen Y (1977) The combined effects of temperature, leaf wetness and inoculum concentration on infection of cucumbers with *Pseudoperonospora cubensis*. Can J Bot 55: 1487.

[9] Neufeld KN, Ojiambo PS (2012) Interactive effects of temperature and leaf wetness duration on sporangia germination and infection of cucurbit hosts by *Pseudoperonospora cubensis*. Plant Dis 96: 345–353. 30727141 10.1094/PDIS-07-11-0560

[10] Fall ML, Van der Heyden H, Beaulieu C, Carisse O (2015) *Bremia lactucae* infection efficiency in lettuce is modulated by temperature and leaf wetness duration under Quebec field conditions. Plant Disease 99: 1010–1019. 30690977 10.1094/PDIS-05-14-0548-RE

[11] Harrison JG (1992) Effects of the aerial environment on late blight of potato foliage—a Review. Plant Pathology 41: 384–416.

[12] Harrison JG, Lowe R (1989) Effects of humidity and air speed on sporulation of *Phytophthora infestans* on potato leaves. Plant Pathology 38: 585–591.

[13] Cohen Y, Perl M, Rotem J (1971) The effect of darkness and moisture on sporulation of *Pseudoperonospora cubensis* in cucumbers. Phytopathology 61: 594–595.

[14] Rotem J, Cohen Y, Bashi E (1978) Host and environmental influences on sporulation *in vivo*. Annu Rev Phytopathol 16: 83–101.

[15] Su H, van Bruggen AHC, Subbarao KV, Scherm H (2004) Sporulation of *Bremia lactucae* affected by temperature, relative humidity, and wind in controlled conditions. Phytopathology 94: 396–401. 10.1094/PHYTO.2004.94.4.396 18944116

[16] Cohen Y, Vaknin M, Ben Naim Y (2015) Chemical control of downy mildew in basil caused by *Peronospora belbahrii*. Phytoparasitica 43: 381.

